# Networked lymphatic endothelial cells in a transplanted cell sheet contribute to form functional lymphatic vessels

**DOI:** 10.1038/s41598-022-26041-0

**Published:** 2022-12-15

**Authors:** Ayumi Inoue Nagahara, Jun Homma, Bikei Ryu, Hidekazu Sekine, Yuhei Higashi, Tatsuya Shimizu, Takakazu Kawamata

**Affiliations:** 1 Department of Neurosurgery, Graduate School of Tokyo Women’s Medical University, 8-1 Kawada-cho, Shinjuku-ku, Tokyo, 162-8666 Japan; 2 Institute of Advanced Biomedical Engineering and Science, TWIns, Tokyo Women’s Medical University, 8-1 Kawada-cho, Shinjuku-ku, Tokyo, 162-8666 Japan; 3 Department of Neurosurgery, Tokyo Women’s Medical University Hospital, 8-1 Kawada-cho, Shinjuku-ku, Tokyo, 162-8666 Japan; 4 Tokaihit Co., Ltd., Shizuoka, Japan

**Keywords:** Biological techniques, Cell biology, Medical research, Engineering

## Abstract

This study evaluated whether cell sheets containing a network of lymphatic endothelial cells (LECs) promoted lymphangiogenesis after transplantation in vivo. Cell sheets with a LEC network were constructed by co-culturing LECs and adipose-derived stem cells (ASCs) on temperature-responsive culture dishes. A cell ratio of 3:2 (vs. 1:4) generated networks with more branches and longer branch lengths. LEC-derived lymphatic vessels were observed 2 weeks after transplantation of a three-layered cell sheet construct onto rat gluteal muscle. Lymphatic vessel number, diameter and depth were greatest for a construct comprising two ASC sheets stacked on a LEC/ASC (3:2 ratio) sheet. Transplantation of this construct in a rat model of femoral lymphangiectomy led to the formation of functional lymphatic vessels containing both transplanted and host LECs. Further development of this technique may lead to a new method of promoting lymphangiogenesis.

## Introduction

Although medical and surgical treatments are available for many neurological disorders, diseases such as stroke, Alzheimer’s disease, traumatic brain injury, multiple sclerosis and Parkinson’s disease remain incurable. Lymphatic vessels play vital roles in the transfer of interstitial fluid, waste products, cytokines and proteins. It is now recognized that lymphatic vessels may be involved in the pathogenesis of a variety of neurological conditions including Alzheimer’s disease, Parkinson’s disease, stroke, cerebral trauma, multiple sclerosis, brain tumors and age-related cognitive decline^1^. Furthermore, there is now interest in exploring whether therapies targeting lymphatic vessels might have potential for development into new treatments for neurological disorders. For example, hydrogels containing vascular endothelial growth factor-C (VEGF-C) were found to increase the diameters of lymphatic vessels in the meninges of aging rats and improve cognitive function^2^. Hence, the development of lymphangiogenesis-inducing therapies might lead to advances in the treatment of various neurological diseases.

Regenerative medicine is a rapidly expanding field, and it is hoped that many diseases that are currently incurable will be treatable in the future using cell-based therapies. Previous studies aimed at developing cell-based therapies have used cell-sheet-based tissue engineering to construct two-dimensional cardiac^3^, pancreatic^4^ and hepatic^5^ tissue. A notable feature of these previous studies is that scaffold-free tissue could be generated using temperature-responsive culture dishes, which allow cellular adhesion at 37 °C but promote cellular detachment at 20 °C. The harvesting of cells from a temperature-responsive culture dish does not require proteases, which helps to preserve the extracellular matrix, cell–cell junctions^6^ and cell adhesion molecules and thereby facilitates tissue engraftment after transplantation^7^. Moreover, cell sheets can be stacked to produce scaffold-free, three-dimensional (3D) tissues with cell adhesion molecules^8^.

Adipose-derived stem cells (ASCs) are considered a desirable source of cells for regenerative medicine and can be engineered into cell sheets. The transplantation of layered ASC sheets has been shown to promote angiogenesis and neurogenesis through paracrine effects and exert beneficial effects in rat models of arterial injury^7^ and stroke^9^. In addition, this growth factor-mediated paracrine effect of ASCs has been demonstrated to enhance the formation of vascular networks when ASCs were co-cultured with blood vessel endothelial cells (ECs)^10^. Sasagawa et al. confirmed that an ASC sheet containing a network of ECs was able to rapidly connect to host vessels during the early post-transplantation period^11^.

Lymphangiogenesis is mediated by lymphangiogenic factors such as VEGF-C, and several previous investigations have described the use of tissue engineering technology to initiate lymphangiogenesis. For example, lymphatic endothelial cells (LECs) were shown to form lymphatic vessel-like structures in 3D collagen and fibrin matrices^12^. Furthermore, 3D hydrogel-based dermo-epidermal skin grafts containing LECs were demonstrated to generate lymphatic vessels, which anastomosed with the host rat’s lymphatic vasculature after the graft was transplanted onto a skin wound^13^. Additionally, a protocol has been described to generate a scaffold-free 3D lymphatic network from co-cultured LECs and fibroblasts^14^. However, no prior studies have reported the formation of lymphatic vessels in transplanted cell sheets that were generated using temperature-responsive culture dishes. The main aims of the present study were to engineer cell sheets containing a network of LECs and evaluate whether the cell sheets promoted lymphangiogenesis after their transplantation onto rat subcutaneous tissue and in a model of muscle tissue lymphangiectomy. We hypothesized that the inclusion of LECs in a cell sheet would promote lymphangiogenesis after transplantation similar to the manner in which the inclusion of ECs enhances angiogenesis in a cell sheet^11^. Therefore, we first co-cultured ASCs and LECs in a cell sheet and established which experimental conditions would favor the creation of a networked LEC sheet. Then, we investigated whether lymphatic vessels developed in the networked LEC sheet after its transplantation.

## Results

### Evaluation of growth factors in the culture medium bathing ASCs

**Figure 1:**
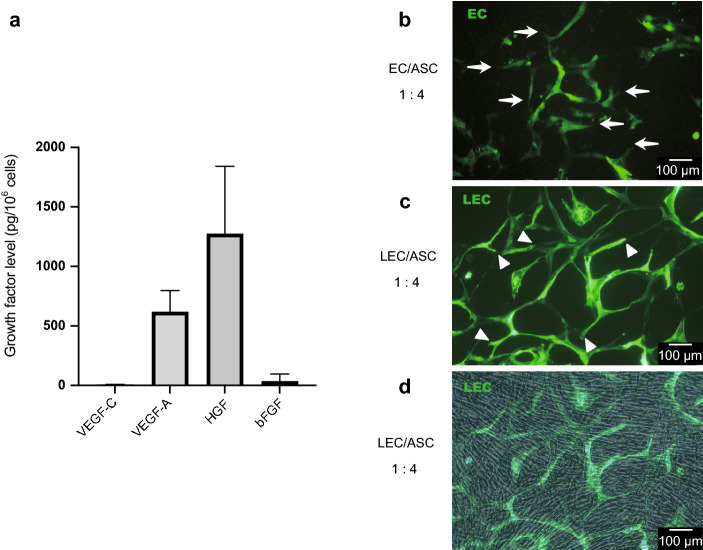
Secretion of growth factors by adipose-derived stem cells (ASCs) and the formation of networks by co-culture with endothelial cells (ECs) or lymphatic endothelial cells (LECs). (**a**) The levels of vascular endothelial growth factor-C (VEGF-C), VEGF-A, hepatocyte growth factor (HGF) and basic fibroblast growth factor (bFGF) in the medium bathing cultured ASCs were measured using enzyme-linked immunosorbent assays. HGF and VEGF-A were both secreted by ASCs, whereas the levels of secreted VEGF-C and b-FGF were much lower. (**b**) Green fluorescent protein (GFP)-expressing ECs and ASCs co-cultured at a ratio of 1:4. Networks of vascular ECs were observed, and the edges of the vascular EC network were sharp (arrows). (**c**) GFP-LECs and ASCs co-cultured at a ratio of 1:4. The LECs formed networks, but in contrast to vascular ECs, rounded/bulging structures were observed at the edges of the LEC network (arrowheads). (**d**) Merge image of (**c**) and phase-contrast image. ASCs shows random pattern with no specific character which do not form own network or support LEC network directly.

Lymphangiogenesis and angiogenesis are stimulated by growth factors, mechanical stimuli or surrounding environment like inflammation or wound healing in lymphangiogenesis. Our initial experiments focused on growth factors evaluated whether ASCs secreted hepatocyte growth factor (HGF), VEGF-A, VEGF-C and basic fibroblast growth factor (bFGF). VEGF-C is chosen to evaluate as lymphangiogenic growth factor. VEGF-A, VEGF-D, FGF-2, HGF and/or Ang-1 are known growth factors secreted by ASCs to stimulate LECs to migrate and form tubular structures, so HGF, VEGF-A and bFGF are chosen to evaluate also^15,16^. HGF and VEGF-A were both secreted into the culture medium by ASCs, whereas the levels of VEGF-C and bFGF were much lower (P < 0.05, one-way ANOVA, Fig. 1a). The secreted HGF level was significantly higher than the secreted VEGF-A, VEGF-C and bFGF levels, and that the secreted VEGF-A level was significantly higher than the secreted VEGF-C and bFGF levels (P < 0.05 for all pairwise comparisons). There was no significant difference in the culture medium levels of VEGF-C and bFGF.

### LECs formed networks when co-cultured with ASCs

Green fluorescent protein-expressing ECs (GFP-ECs) formed networks when co-cultured with ASCs (1:4 cell ratio), and the edges of the vascular network were pointed (Fig. 1b). LECs co-cultured with ASCs (1:4 ratio) also formed a network, but the structures at the edges of the LEC network formed bulging structures like blind-ended sacs (Fig. 1c). LEC network was confirmed by co-culturing LEC with ASC by 1:4 cell ratio. Similar to this, EC network was confirmed by co-culturing EC with ASC by 1:4 cell ratio. Merge image of co-culture of GFP-LECs with ASCs and phase contrast image show ASCs do not compose network within ASCs nor directly support LEC network since it is random pattern without any specific character observed (Fig. 1d).

### Effects of cell ratio and VEGF-C concentration on the mesh pattern of LECs co-cultured with ASCs

**Figure 2:**
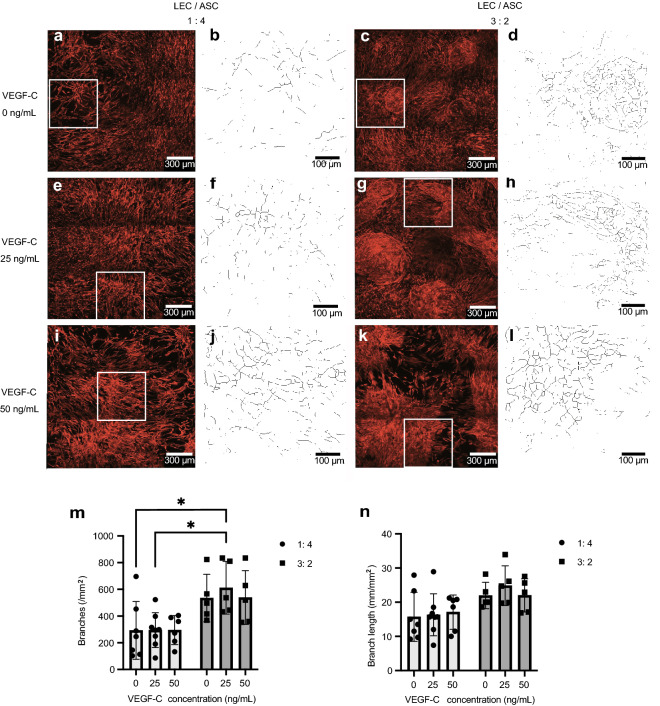
Effects of cell ratio and vascular endothelial growth factor-C (VEGF-C) concentration on the mesh pattern by lymphatic endothelial cells (LECs) co-cultured with adipose-derived stem cells (ASCs). (**a–l**) Representative images of LECs co-cultured with ASCs and immunostained for podoplanin (a glycoprotein expressed by LECs). The LECs were cultured in medium containing 0 ng/mL VEGF-C (**a**–**d**), 25 ng/mL VEGF-C (**e**–**h**) or 50 ng/mL VEGF-C (**i**–**l**), and the ratio of LECs to ASCs was either 1:4 (**a**,**b**,**e**,**f**,**i**,**j**) or 3:2 (**c**,**d**,**g**,**h**,**k**,**l**). High-power images of (**a**,**c**,**e**,**g**,**i**,**k**) which were skeletonized by ImageJ software to evaluate branches and branch lengths are shown in (**b**,**d**,**f**,**h**,**j**,**l**). LECs co-cultured with ASCs at a ratio of 1:4 in 0 ng/mL VEGF-C or 25 ng/mL VEGF-C generated elongated structures but did not form ‘islands’ (**a**,**b**,**e**,**f**). Similar results were obtained when LECs were co-cultured with ASCs at a ratio of 1:4 in 50 ng/mL VEGF-C, although the LEC mesh pattern was denser than that obtained in the lower concentrations of VEGF-C (**i**,**j**). LECs co-cultured with ASCs at a ratio of 3:2 in 0 ng/mL VEGF-C formed island-like structures with a dense LEC mesh pattern (**c**,**d**). LECs co-cultured with ASCs at a ratio of 3:2 in 25 ng/mL VEGF-C or 50 ng/mL VEGF-C formed more prominent island-like structures containing a dense and fine LEC mesh pattern (**g**,**h**,**k**,**l**). (**m**) Density of branches in the LEC mesh pattern (number of branches per mm^2^). Significantly more branches were observed for a LEC/ASC ratio of 3:2 than for a ratio of 1:4 (P < 0.0001, two-way analysis of variance [ANOVA]). However, the concentration of VEGF-C had no significant effect on junction formation (P = 0.83, two-way ANOVA). Pairwise comparisons showed that branch density in the 3:2 ratio/25 ng/mL VEGF-C group was significantly higher than that in the 1:4 ratio/0 ng/mL VEGF-C group or the 1:4 ratio/25 ng/mL VEGF-C group (P < 0.05). All conditions were quantified and compared. Non-significant differences were not denoted on the graphs. The data are presented as the mean ± standard deviation (n = 8 per group). * P < 0.05. (**n**) LEC mesh pattern branch length (mm/mm^2^). Branch length was longer for a LEC/ASC ratio of 3:2 than for a ratio of 1:4 (P = 0.002, two-way ANOVA). However, VEGF-C had no significant effect on branch length (P = 0.76, two-way ANOVA). All conditions were quantified and compared. Non-significant differences were not denoted on the graphs. The data are presented as the mean ± standard deviation (n = 8 per group).

Representative images of LECs co-cultured with ASCs and immunostained for podoplanin (a glycoprotein expressed by LECs) are shown in Fig. 2a–l. VEGF-C concentration had little or no effect on the mesh pattern by LECs, whereas altering the LEC/ASC ratio from 1:4 to 3:2 appeared to promote the formation of ‘islands’ containing dense mesh pattern of LECs (Fig. 2a–l). Quantitative evaluations revealed that VEGF-C concentration had no significant effect on the number of branches per mm^2^ (Fig. 2m) or the branch length per mm^2^ (Fig. 2n). However, changing the LEC/ASC ratio from 1:4 to 3:2 was associated with more branches per mm^2^ (P < 0.05, two-way ANOVA, Fig. 2m) and a longer branch length per mm^2^ (P < 0.05, two-way ANOVA, Fig. 2n). Pairwise comparisons showed that branch density in the 3:2 ratio/25 ng/mL VEGF-C group was significantly higher than that in the 1:4 ratio/0 ng/mL VEGF-C group or the 1:4 ratio/25 ng/mL VEGF-C group (P < 0.05).

### Lymphatic vessel-like structures were evident in a three-layered LEC/ASC cell sheet (1:4 ratio) 2 weeks after transplantation onto rat gluteal muscle

**Figure 3:**
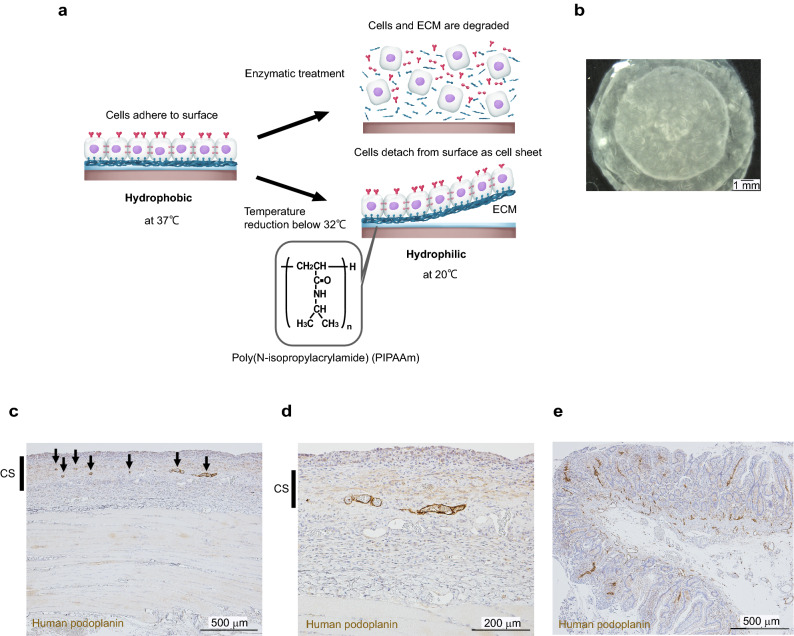
Lymphatic vessel-like structures were evident in a three-layered LEC/ASC sheet (1:4 ratio) 2 weeks after transplantation onto rat gluteal muscle. (**a**) Temperature-responsive culture dish coated with Poly(*N*-isopropylacrylamide) (PIPAAm), temperature-responsive polymer used to construct cell sheet. At temperature of 37 °C, cells adhere to surface of dish, and temperature decreased to 20 °C enable cells detach from dish preserving their cell–cell junctions, cell surface proteins and extracellular matrix without need of enzymatic treatment which breaks cell–cell junctions. (**b**) Observation of three cell sheets stacked before implantation by stereo microscope. (**c**) Three LEC/ASC sheets were stacked and transplanted onto rat gluteal muscle in vivo, and immunostaining for podoplanin was performed 2 weeks later. The low-power image shows LECs positive for human podoplanin. *CS* cell sheet. (**d**) High-power image showing a few lymphatic vessel-like structures composed of LECs. (**e**) Positive control image of human podoplanin in human small intestine.

To explore whether lymphatic vessels would develop in LEC/ASC sheets after transplantation in vivo, three LEC/ASC sheets (1:4 ratio) were stacked (Fig. 3c) and transplanted onto rat gluteal muscle. It is a first stage proof-of-concept to confirm LEC/ASC sheets can organize lymphatic vessel-like structures in vivo. Immunostaining of the cell sheets/underlying gluteal muscle for podoplanin 2 weeks after transplantation revealed the presence of vessel-like structures composed of LECs (Fig. 3c), and high-power images confirmed that corpuscles had migrated within the cell sheets (Fig. 3d). Development of lymphatic vessel-like structures by cell sheet transplantation were observed.

### Comparison of LEC-derived lymphatic vessels between different methods of cell transplantation onto rat gluteal muscle

**Figure 4:**
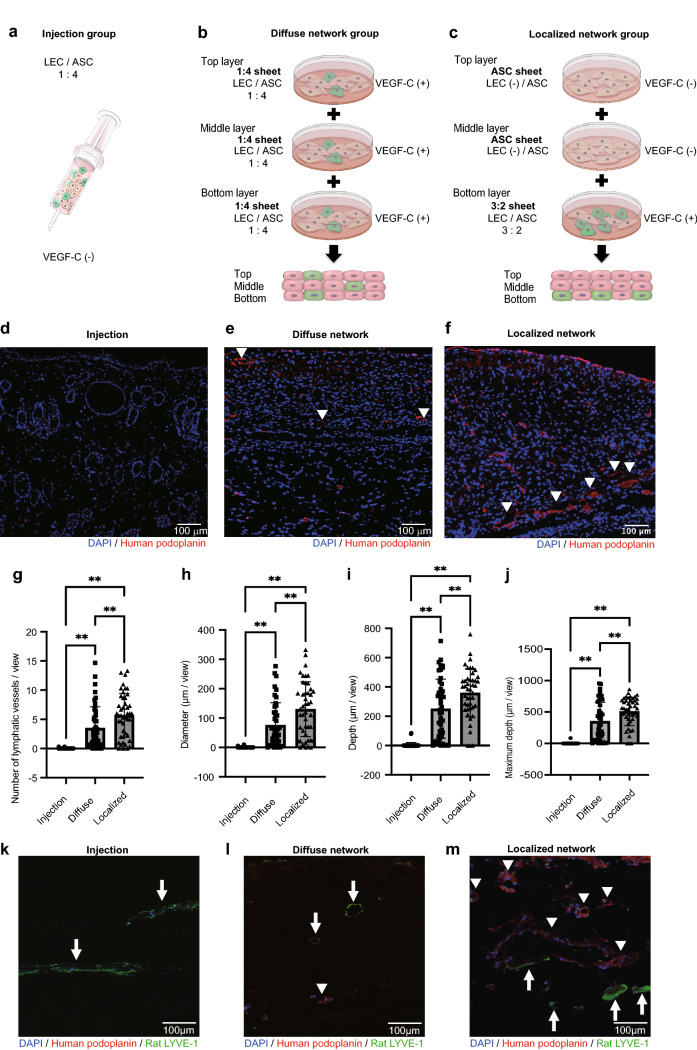
Comparison of lymphatic vessel-like structures between different methods of cell transplantation onto rat gluteal muscle. (**a**) A total of 0.6 × 10^6^ LECs and 2.4 × 10^6^ ASCs were collected in 100 μL of phosphate-buffered saline for injection. (**b**) In the diffuse network group, three LEC/ASC sheets (1:4 ratio) were stacked for transplantation. (**c**) In the localized network group, two ASC sheets were stacked on one LEC/ASC sheet (3:2 ratio) for transplantation. (**d–f**) Tissue sections immunostained for podoplanin. Nuclei were counterstained with DAPI (blue). The arrowheads show lymphatic vessel-like structures derived from the transplanted lymphatic endothelial cells (LECs). No lymphatic vessel-like structures were observed in the cell injection group; however, host vessels had formed due to inflammation (**d**). (**g–j**) Number (**g**), diameter (**h**), average depth (**i**) and maximum depth (**j**) of lymphatic vessel-like structures staining positively for human podoplanin. The data are presented as the mean ± standard deviation (n = 5 per group). **P < 0.01. (**k–m**) Tissue sections immunostained for human podoplanin (red) and rat LYVE-1 (green). Nuclei were counterstained with DAPI (blue). The arrowheads show lymphatic vessel-like structures derived from the transplanted lymphatic endothelial cells (LECs). No lymphatic vessel-like structures were observed in the cell injection group; however, arrows show appearance of host lymphatic vessels (**k**). Host lymphatic vessels (arrows) and graft lymphatic vessel-like structures (arrowheads) were observed separately in diffuse network group (**l**). Connection of a host LEC with graft lymphatic vessel-like structure was observed in localized network group (**m**).

In the next series of experiments, we compared three different methods of cell transplantation onto rat gluteal muscle: injection of a suspension of LECs and ASCs at a 1:4 ratio (cell suspension group, Fig. 4a); transplantation of three stacked LEC/ASC (1:4 ratio) sheets (diffuse LEC network group, Fig. 4b); and transplantation of two ASC sheets stacked together with one LEC/ASC (3:2 ratio) sheet (localized LEC network group, Fig. 4c). Immunostaining for human podoplanin was used to identify lymphatic vessel-like structures derived from the transplanted LECs (Fig. 4d–f), and the characteristics of the lymphatic vessels derived from the transplanted LECs were quantified (Fig. 4g–j). The cell suspension group contained a larger number of host vessels (i.e., arterial, venous and lymphatic vessels of host that were negative for anti-human podoplanin antibody) in the subcutaneous layer than the cell sheet groups, which may have been due to an inflammatory reaction to the injection (Fig. 4d). At 1 week after transplantation, lymphatic vessels positive for human podoplanin were absent in the cell suspension group (Fig. 4d), occasionally seen in the diffuse LEC network group (Fig. 4e), and more frequently observed in the localized LEC network group (Fig. 4f). The number, average diameter, average depth and maximal depth of the lymphatic vessel-like structures were all significantly higher in the diffuse LEC network group than in the cell suspension group (P < 0.01 for all parameters; Fig. 4g–j). Furthermore, the number, average diameter, average depth and maximal depth of the lymphatic vessel-like structures were significantly higher in the localized LEC network group than in the cell suspension group (P < 0.01 for all parameters) or diffuse LEC network group (P < 0.01 for all parameters; Fig. 4g–j). Therefore, the number, diameter and depth of the LEC-derived lymphatic vessels were greater for the localized LEC network group than for the other groups.

Focusing on relation between host lymphatic vessels and graft lymphatic vessel-like structures, there were only host lymphatic vessels confirmed in injection group (Fig. 4k), host lymphatic vessels and graft lymphatic vessel-like structures were observed separately in diffuse LEC network group (Fig. 4l), and connection of host LEC and graft lymphatic vessel-like structure were observed in localized LEC network group (Fig. 4m).

### Transplantation of cell sheets with a localized LEC network assisted lymphatic vessel reconstruction in a rat model of femoral lymphangiectomy

**Figure 5:**
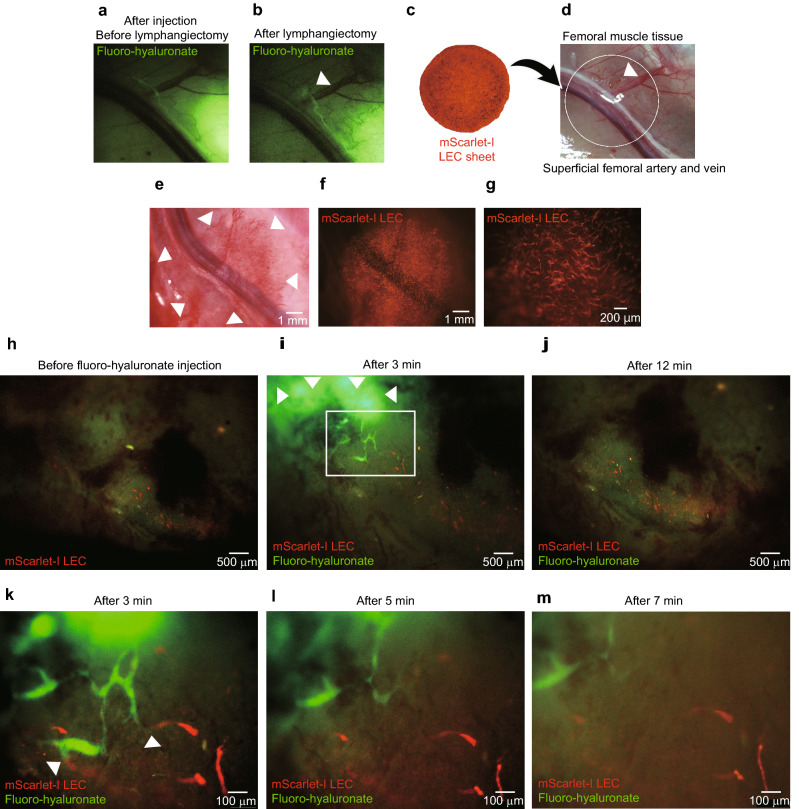
Transplanted cell sheets containing a localized network of mScarlet-I-labeled LECs contribute to the reconstruction of functional lymphatic vessels in a rat model of femoral lymphangiectomy. (**a**) Confirmation of lymphatic vessel appear alongside to superficial femoral artery and vein in femoral muscle by injecting fluorescent hyaluronic acid (fluoro-hyaluronate) in distal femoral muscle. (**b**) Leakage of fluoro-hyaluronate was observed after lymphangiectomy by resecting femoral muscle tissue. (**c**) A cell sheet containing a localized network of mScarlet-I-labeled LECs. (**d**) A rat model of femoral lymphangiectomy was generated by resecting a 1.5 × 1.5 mm piece of femoral muscle tissue including its lymphatic vessels. Cell sheets containing a localized network of mScarlet-I-labeled LECs were transplanted onto the defect. The white circle indicates the region where the cell sheets were transplanted. (**e**) Macroscopic image of the femur 1 week after transplantation of the cell sheet construct. Neovascularization was evident in the region of the muscle surface where the cell sheets had been transplanted (arrowheads). (**f**) Macroscopic fluorescence observation of mScarlet-I-labeled LECs. (**g**) Magnified image showing mScarlet-I fluorescence. A reticular network was evident that was similar to that seen before transplantation. (**h–m**) The ability of the lymphatic vessels to take up substances from the interstitium was evaluated by injecting fluoro-hyaluronate. Only the red signal of the mScarlet-I-labeled lymphatic endothelium was observed before the injection of fluoro-hyaluronate (**h**). A green signal in the lymphatic vessels was observed 3 min after the injection of fluoro-hyaluronate (arrowheads indicate the interstitial area), demonstrating that the host lymphatic vessels had taken up hyaluronic acid from the interstitium (**i**). The green signal in the lymphatic vessels disappeared after 12 min, implying that the lymphatic vessels had circulated the fluoro-hyaluronate away from the region of observation (**j**). The magnified image (**k–m**) of the region marked by the white rectangle in (**i**) shows connections between the host lymphatic vessels and the transplanted lymphatic vessels at 3 min after fluoro-hyaluronate injection (**k**, arrowheads). The green signal gradually disappeared over the subsequent 4 min (**l**, **m**), which would be consistent with the flow of lymph fluid away from the region of observation.

A rat model of femoral lymphangiectomy was generated by resecting a 1.5 × 1.5 mm piece of femoral muscle and its lymphatic vessels, and cell sheets containing a localized network of mScarlet-I-labeled LECs were transplanted onto the defect (Fig. 5a–d). Observation of the transplantation site 1 week after transplantation of the cell sheet revealed neovascularization on the surface of the muscle layer where the graft had been implanted (Fig. 5e). In addition, a network of LECs was observed in this region (Fig. 5f,g). The function of the lymphatic vessels was evaluated by injecting fluorescent hyaluronic acid into the interstitium: a fluorescence signal appeared in the host lymphatic vessels from 3 min after the injection of fluorescent hyaluronic acid and disappeared after 12 min, indicating that the host lymphatic vessels were able to capture hyaluronic acid from the interstitium and circulate it (Fig. 5h–j). Furthermore, observations made at high magnification between 3 and 7 min after hyaluronic acid injection (Fig. 5k–m) demonstrated connections (arrowheads) between the host lymphatic vessels and the transplanted lymphatic endothelium. The above findings suggest that transplanted LECs may contribute to lymphatic vessel reconstruction.

### Reconstruction of lymphatic vessel morphology after the transplantation of cell sheets containing a localized network of LECs

**Figure 6:**
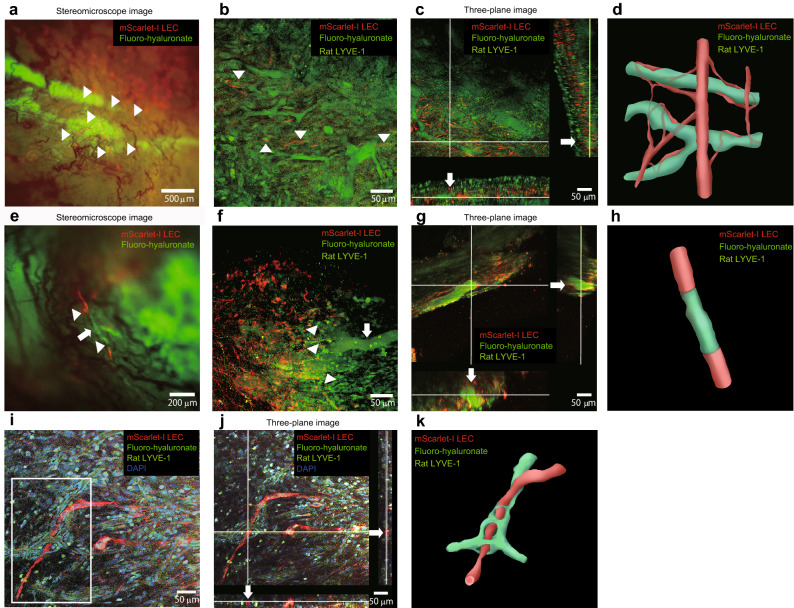
Analysis of the direct contribution of transplanted cells to lymphatic vessel reconstruction using a two-photon laser microscopy system. (**a**) Stereoscopic fluorescence microscopy image of a cell sheet containing a localized network of LECs obtained 1 week after transplantation. Interactions (arrowheads) are evident between fluoro-hyaluronate-containing host lymphatic vessels (green) and the transplanted LEC network (red). (**b**) Image of the structure in (**a**) obtained by two-photon laser microscopy after tissue clearing. The arrowheads show regions where transplanted LECs (red) formed parts of the walls of host lymphatic vessels (immunostained for rat LYVE-1; green) that had taken up fluoro-hyaluronate (green). (**c**) Three-dimensional two-photon laser microscopy of the structure in (**b**) after tissue clearing (three-plane image). At the intersection of the white lines, transplanted LECs (red) were observed in the wall of a host lymphatic vessel (arrows) that contained fluoro-hyaluronate (green). (**d**) Stereofluorescence microscopy image of a cell sheet construct with a localized network of LECs at 1 week after transplantation. The arrowheads show end-to-end connections between a fluoro-hyaluronate-containing host lymphatic vessel (green) and transplanted lymphatic endothelium (red). (**e**) Image of the structure in (**d**) acquired with two-photon laser microscopy after tissue clearing. mScarlet-I-labeled LECs partially co-localized with rat LYVE-1-positive host lymphatic vessels that contained fluoro-hyaluronate, suggesting that the transplanted LECs formed part of the lymphatic vessel wall (arrowheads). (**f**) Three-plane image of the structure in (**d**) captured by two-photon laser microscopy after tissue clearing. At the intersection of the white lines, the fluoro-hyaluronate-containing lymphatic vessels (green) were observed to have a vascular wall composed of transplanted LECs (red) (arrows). Yellow signals indicate anastomoses of rat LECs and transplanted human LECs composing functional lymphatic vessels (arrows). (**g**) Two-photon laser microscopy image obtained after tissue clearing. The transplanted mScarlet-I-labeled LECs (red) were connected to each other as a chain of cells. The region in the white rectangle shows the newly formed human lymphatic vessel (comprising mScarlet-I-labeled LECs) intertwining with the lymphatic vessels of the host rat (immunostained for rat LYVE-1). Cell nuclei are stained with DAPI. (**h**) Three-plane image of the structure in (**g**) obtained by two-photon laser microscopy after tissue clearing. At the intersection of the white lines, the transplanted human LECs (red) formed a vessel with a lumen (arrows). (**i**) Illustration of the morphology of the structure in (**g**). The lymphatic vessels of the host rat are shown in green, and the vessel formed by the transplanted human LECs is shown in red. Remodeling of the lymphatic vessels had resulted in the vessels intertwining with each other.

Two-photon laser scanning microscopy was used for 3D structure analysis of mScarlet-I-labeled LECs and host lymphatic vessels immunostained for rat lymphatic vessel hyaluronan receptor-1 (LYVE-1) at 1 week after the transplantation of cell sheets containing a localized network of LECs. Stereofluorescence microscopy of the tissue surface 1 week after transplantation of the cell sheets revealed intersections between hyaluronate-incorporated host lymphatic vessels and transplanted LECs (Fig. 6a). Two-photon laser scanning microscopy of this structure showed that transplanted LECs formed parts of the walls of host lymphatic vessels that had taken up fluoro-hyaluronate (Fig. 6b). In addition, three-dimensional depth images (three-plane images) of this structure obtained by two-photon laser microscopy showed that the walls of lymphatic vessels, which had taken up fluoro-hyaluronate, contained transplanted LECs at the intersection of the white lines (Fig. 6c). In other sample of the localized network group, stereofluorescence microscopy showed end-to-end connections between hyaluronate-containing host lymphatic vessels and transplanted LECs (Fig. 6e). Two-photon laser scanning microscopy of this structure confirmed the presence of end-to-end junctions between transplanted LEC-covered vessels and a hyaluronate-containing lymphatic vessel (Fig. 6f). Three-dimensional depth images (three-plane images) of this structure confirmed that the wall of the lymphatic vessel, which had taken up fluoro-hyaluronate, was composed of transplanted LECs at the intersection of the white lines (Fig. 6g). Two-photon laser microscopy of another sample observed transplanted LECs as several interconnected and elongated cells (Fig. 6i). A unique relationship between transplanted human LECs (labeled with mScarlet-I) and host lymphatic vessels (immunostained for rat LYVE-1) is shown in the white rectangle in Fig. 6i. Three-dimensional depth images (three-plane images) revealed that the transplanted human LECs formed a luminal structure at the intersection of the white lines (Fig. 6j). An illustration depicting the morphological relationship between the transplanted LECs and host lymphatic vessel is shown in Fig. 6k. These findings indicate that transplanted human LECs may play a variety of reconstruction-related roles, such as: (1) fusing with host lymphatic vessels and contributing to host lymphatic vessel reconstruction (Fig. 6d); (2) forming independent vessels that connect to host lymphatic vessels (Fig. 6h); and (3) forming lymphatic vessels that are independent of the host lymphatic vessels (Fig. 6k).

## Discussion

We successfully established a networked LEC sheet and achieved reconstrcution of lymphatic vessels in a rat model of lymphangiectomy after the transplantation of these cell sheets. To the best of our knowledge, this is the first study to demonstrate lymphangiogenesis after the transplantation of cell sheets constructed using temperature-responsive culture dishes. In addition to confirming that the LEC network in a cell sheet could generate lymphatic vessels, we found that creation of a LEC network was favored by a higher concentration of LECs relative to ASCs. Moreover, a cell sheet construct with a high-density LEC network (localized network group) generated lymphatic vessel-like structures that were greater in number, diameter and depth than those generated by cell sheets with a lower-density network (diffuse network group).

This study established a networked LEC sheet by co-culturing LECs with ASCs in conditioned endothelial basal medium-2 (EBM-2 medium), which is appropriate for ECs. EBM-2 medium consists of human epidermal growth factor, VEGF-A, R3-insulin-like growth factor-1, ascorbic acid, hydrocortisone, human fibroblast growth factor-beta, fetal bovine serum (FBS) and gentamicin/amphotericin-B. Notably, this medium does not contain VEGF-C, which is known to be an important growth factor driving lymphangiogenesis^17^. However, the present study revealed that the addition of VEGF-C (25 or 50 ng/mL) did not improve the mesh pattern of a LEC in conditioned EBM-2 medium in vitro. This finding suggests the involvement of other lymphangiogenic growth factors present in the EBM-2 medium and/or secreted by ASCs. Furthermore, a higher concentration of LECs relative to ASCs favored mesh pattern in vitro, as evidenced by the higher branch density and longer branch length for LECs/ASCs co-cultured at ratio of 3:2 than for cells co-cultured at a ratio of 1:4. LECs/ASCs co-cultured at a 3:2 ratio formed ‘islands’ containing dense mesh pattern of LEC, whereas cells co-cultured at a 1:4 ratio rarely formed ‘islands’ but instead generated more sparse mesh pattern (Fig. 2). Moreover, lymphatic vessels were successfully reconstructed in vivo after the transplantation of networked LEC sheets but not after the injection of a cell suspension.

In this study, LECs were co-cultured with ASCs because ASCs are known to support vessel formation by ECs in vitro and in vivo and enhance the migration, proliferation and tube formation of LECs either directly or via the secretion of growth factors^18^. Knezevic et al. reported that LECs cultured without ASCs or VEGF-C did not form networks, and only cell elongation was observed when culture was performed in the absence of VEGF-C^19^. Network formation also did not occur when LECs were cultured in unconditioned medium for ASCs or unconditioned EBM-2 medium. Notably, increasing concentrations of VEGF-C (from 0 to 50 ng/mL) enhanced the densities of the networks formed by LECs when co-cultured with ASCs on fibrin hydrogels. Another study by Robering et al. reported proliferation and tube formation of LEC were achieved by conditioned medium of mesenchymal stem cells as same or even higher level as combination of VEGF-C and bFGF^20^.

In the present study, LECs co-cultured with ASCs in conditioned EBM-2 medium formed mesh pattern at cell ratios of 3:2 and 1:4, with a cell ratio of 3:2 producing mesh pattern with more branches and longer branch lengths. However, increasing the VEGF-C concentration from 0 to 50 ng/mL had little effect on mesh pattern at either cell ratio, which is not consistent with the results of Knezevic et al.^19^. One possible explanation for the apparent inconsistency is that Knezevic et al. utilized a fibrin hydrogel for cell culture. Takeda et al.^21^ reported that the effects of increasing the VEGF-C concentration (from 0 to 100 ng/mL) were not as great as the effect of co-culturing with ASCs, which agrees with the findings of the present study. It is possible that the effectiveness of VEGF-C on LEC mesh pattern may depend on whether the LECs are co-cultured and the type of substrate used. Irrespective, the present findings show that LECs can generate a mesh pattern when they are co-cultured with ASCs in conditioned EBM-2 medium and that a higher concentration of LECs to ASCs favors the formation of a dense mesh pattern in vitro.

Lymphangiogenesis occurs in response to inflammation, wound healing or tumor regression, and LECs are affected by several types of lymphangiogenic factors such as VEGF-A, VEGF-C, VEGF-D, FGF-2, HGF, insulin-like growth factor-1 (IGF-1), IGF-2, angiopoietin-1 (Ang-1), Ang-2 and platelet-derived growth factor-bb (PDGF-BB)^19^. Furthermore, the mRNAs for VEGF-A, VEGF-C, VEGF-D, FGF-2, HGF, Ang-1 and IGF-1 are expressed in ASCs. Hsiao et al. and Strassburg et al. suggested that ASCs stimulate LECs to migrate and form tubular structures either directly or by secreting growth factors such as VEGF-A, VEGF-D, FGF-2, HGF and/or Ang-1^15,16^. However, debate remains as to whether VEGF-C is secreted by ASCs co-cultured with ECs. Therefore, the present study evaluated whether VEGF-A, VEGF-C, HGF and bFGF were secreted by cultured ASCs. The analysis revealed that HGF and (to a lesser extent) VEGF-A were secreted by ASCs, whereas the secretion of VEGF-C and bFGF was low. This result is similar to that obtained by Rehman et al.^22^, who reported that HGF was the main growth factor secreted by ASCs under normoxic conditions followed by VEGF-A and transforming growth factor-beta (TGF-β), whereas there was little or no secretion of granulocyte macrophage colony-stimulating factor or bFGF. In addition, Takeda et al.^21^ evaluated the relative mRNA expressions of lymphangiogenic factors such as VEGF-A, VEGF-C, VEGF-D, FGF-2, HGF, Ang-1 and IGF-1 in ASCs cultured in various media (Dulbecco’s modified Eagle medium [DMEM] with FBS, EGM-2-MV, DMEM or EBM-2). The study found that the expressions of VEGF-C, VEGF-D, HGF, Ang-1 and IGF-1 were increased by serum starvation, suggesting that ASCs adapt their secretion of growth factors to the surrounding environment^21^. The highest expression of VEGF-C was observed when ASCs were cultured in medium appropriate for ECs, which may underlie the effects of ASCs on the differentiation of ECs. The growth factors secreted by ASCs acted synergistically in combination with other growth factors to promote lymphangiogenesis, and this lymphangiogenic effect was stronger than that of VEGF-C. Nevertheless, the measurement of growth factors in the present study differed from that performed in these previous investigations, which collected and evaluated the culture medium after 3 days of culture without replacement of the medium.

This study evaluated growth factor secretion by ASCs in DMEM/nutrient mixture F-12 (DMEM/F12) medium so as to avoid the effects of growth factors found in other culture media. However, LECs were co-cultured with ASCs in EBM-2 medium, which is appropriate for ECs, and the use of this medium may have affected growth factor secretion by the ASCs or differentiation of the ASCs. HGF and VEGF-A were the main growth factors (among those investigated) secreted by ASCs in DMEM/F12 medium. Cabral et al. (2018) reported that HGF interacts with five angiogenic factors, namely VEGF-A, Ang-2, interleukin-8 (IL-8), VEGF-C and endothelin-1 (ET-1), while heparin binding-EGF-like growth factor (HB-EGF) interacts with VEGF-A, IL-8, VEGF-C, ET-1 and follistatin^23^. The EBM-2 medium used in the present study contained HB-EGF, which may have interacted with other growth factors as described above. Importantly, VEGF-A influences Ang-2, IL-8 and ET-1, and VEGF-A may also have direct effects on lymphangiogenesis via the VEGF receptor-2 (VEGFR-2) on LECs as well as indirect effects via the VEGFR-1 on macrophages, which express VEGF-C. Thus, in the present study, VEGF-A and HGF likely played key roles in promoting lymphangiogenesis. This study did not evaluate whether ASCs secreted other growth factors such as VEGF-D, IGF-1, IGF-2, Ang-1, Ang-2, PDGF-BB and EGF, either in DMEM/F12 medium or EBM-2 medium, so it is possible that other growth factors may have contributed to the lymphangiogenic effect. In addition, IL-8 is a known cytokine secreted by ASCs that promotes lymphangiogenesis directly by affecting LECs without activating VEGF-C signaling^21^. Moreover, VEGF-A, HGF and HB-EGF also interact with IL-8, and this interaction may have been an important mechanism promoting lymphangiogenesis in the present study. We speculate that co-culturing LECs with ASCs in EBM-2 medium and combining the cells within a sheet for transplantation are critical factors that allow ASCs to secrete multiple lymphangiogenic factors that induce lymphangiogenesis and the formation of a structured lymphatic endothelial network and lymphatic vessels.

Another study demonstrated that stimulating ASCs with VEGF-C for 48 h in vitro prior to implantation increased the expressions of VEGF-A, VEGF-C and prospero homeobox protein-1 (Prox-1) and enhanced the proliferation of ASCs^24^. These effects were potentiated by the inhibition of TGF-β1 signaling. In addition, stimulation with VEGF-C for 48 h increased the number of podoplanin-positive cells, and this effect was maintained for 10 days, whereas the expressions of Prox-1 and LYVE-1 were maintained only for the first 4 days^24^. The present study also performed experiments in which VEGF-C was added for 48 h prior to the transplantation of LEC/ASC sheets, and an anti-podoplanin antibody was used for evaluation. Although the addition of VEGF-C to cells in vitro did not seem to enhance LEC mesh pattern in the present study, it remains possible that VEGF-C may have stimulated the secretion of growth factors by ASCs, the proliferation of ASCs and the expression of podoplanin-positive cells after transplantation in vivo.

LEC network formation may be supported by the matrix like polyethylene glycol (PEG)^25^, polylactide-co-glycolide (PLGA)^26^, fibrin^26^ or hyaluronic acid^27^ according to previous studies. ASCs secreting matrix is not confirmed so far, however TGF-β1 secreted from ASCs may play important role of constructing matrix to support LEC network formation as collagen deposition and hyaluronic acid synthesis^28^.

Cell sheets generated by the co-culture of LECs and ASCs at a ratio of 3:2 contained three times more LECs than cell sheets generated using a cell ratio of 1:4. Therefore, the three-layered cell sheet constructs transplanted in vivo (diffuse network group and localized network group) were engineered to have the same total number of LECs and ASCs. Although the results of the two-way ANOVA indicated that the cell ratio had a significant effect on LEC mesh pattern whereas the concentration of VEGF-C did not, the only statistically significant difference between the 1:4 group and 3:2 group was observed at a VEGF-C concentration of 25 ng/mL. Therefore, the cell sheets used for in vivo transplantation were generated using a VEGF-C concentration of 25 ng/mL. The in vivo experiments confirmed that cell sheets with a higher local concentration of LECs and a dense network of LECs promoted lymphangiogenesis. The positioning of the sheets within a multi-layered cell sheet is an important factor affecting vessel formation. For example, Asakawa et al.^29^ cultured multi-layered cell sheets on a fibrin gel and compared different configurations made by stacking two normal human dermal fibroblast (NHDF) sheets with one human umbilical vein EC sheet, which was placed in various positions, or by stacking three EC/NHDF co-cultured sheets. Pre-vascular networks composed of ECs were observed in all types of cell sheet. However, a large tubular structure was only observed when the EC sheet was placed on the host side as the bottom layer, indicating that positioning of the ECs in the 3D tissue was an important factor influencing vascular formation. Although the present study utilized LECs and ASCs instead of ECs and NHDFs and evaluated the formation of lymphatic vessels rather than blood vessels, the results were similar to those of Asakawa et al.^29^ in that vessel regeneration was affected by spatial factors and cell polarity, i.e., combining one high-density LEC-containing sheet on the host side with two ASC sheets (localized LEC network group) produced the largest number of lymphatic vessels, the widest lymphatic vessels and the deepest lymphatic vessels. The diffuse LEC network group (three layered cell sheets made using a 1:4 cell ratio) was also able to form lymphatic vessels, but these were fewer in number, smaller in diameter and reached a lesser depth (Fig. 4g–j). By contrast, few lymphatic vessels formed after the injection of a cell suspension (Fig. 4d,g); likely reasons for this include poor cell survival and engraftment after the injection due to physical damage, primary hypoxia and cell wash-out via the bloodstream^30^.

A temperature-responsive culture dish promotes cellular adhesion at 37 °C and cellular detachment at 20 °C, which allows harvesting of a cell sheet without the use of protease. From previous studies regarding cell sheet composed by temperature-responsive dish, fibronectin matrix is known to deposit on the basal surface of cell sheets. Fibronectin matrix plays a key role as an adhesive agent to promote rapid and intimate connections between grafts and hosts^6^. As a result, sheets containing a LEC network can be harvested while preserving their extracellular matrix, cell–cell junctions and cell adhesion molecules, which would be expected to facilitate engraftment after transplantation. No inflammation besides initial and temporally inflammation by surgery procedures is caused in cell sheet transplantation^31^. Lymphangiogenesis after transplantation of the cell sheet construct was likely promoted by activation of ECs by cell–cell interactions (either within the cell sheet or between the cell sheet and host) and paracrine effects mediated by growth factors secreted from ASCs in the sheet.

This study confirmed that lymphatic vessel-like structures were reconstructed after subcutaneous transplantation of cell sheets containing a network of LECs. To evaluate the mechanisms underlying lymphatic vessel reconstruction and the functionality of the new vessels, we transplanted cell sheets (localized network group) into a rat model of femoral lymphangiectomy and analyzed whether human LECs contributed to lymphatic vessel reconstruction in the host. Lymphatic vessels were visualized 3 min after the injection of fluoro-hyaluronate (green signal) into the tissue stroma (Fig. 5i,k), and the fluoro-hyaluronate fluorescence signal disappeared after 12 min (Fig. 5m). These findings confirm that fluoro-hyaluronate can be used to evaluate the uptake of substances from the interstitium by lymphatic vessels. Analysis of the relationship between host lymphatic vessels and transplanted human LECs showed that they overlapped with each or were joined together (arrowheads in Fig. 6a,e), suggesting that morphological connections had been made. Detailed three-dimensional observations with a two-photon laser microscope revealed that the walls of the constructed lymphatic vessels were composed of host lymphatic vessels (rat LYVE-1-positive cells) and some transplanted cells (mScarlet-I-labeled LECs) (Fig. 6b,f). Some vessels containing fluoro-hyaluronate were found to have walls comprising both transplanted cells (mScarlet-I-labeled LECs) and host cells (rat LYVE-1-positive cells) (Fig. 6c,g), indicating that reconstructed lymphatic vessels composed of transplanted LECs and host LECs were capable of taking up tissue fluid (an important function of lymphatic vessels). The results of this study suggest that, when transplanted in the form of a cell sheet with a localized network, human LECs can: (1) fuse with and contribute to the reconstruction of host lymphatic vessels (Fig. 6d); (2) form luminal structures of their own that join with host lymphatic vessels (Fig. 6h); and (3) develop their own lymphatic vessels while intertwining with host lymph vessels in complex ways (Fig. 6k). Nor have we been able to evaluate whether the reconstructed lymphatic vessels are connected to the host veins and lymphatic vessels.

Reconstruction of lymphatic vessels can potentially be a novel therapeutic treatment of diseases associated with lymphatic vessel impairment in which novel functional roles of the lymphatic vessel are identified, such as obesity, cardiovascular disease, atherosclerosis, myocardial infarction, neurological disorders, neurodegenerative disease, lymphatics in ocular disease and lymphatics in inflammatory bowel diseases.

Previous studies in tissue engineering field have also achieved lymphangiogenesis such as using dermo-epidermal skin grafts containing lymph and blood plexus constructed by human LECs and human blood vascular endothelial cells in a fibrin-collagen gel^14^, establish of lymphatic vascularization by implanted LECs and bone marrow-derived mesenchymal stem cells in a fibrin gel surrounding the arteriovenous loop^32^, or in vivo using connective tissue substitute which required 31 days to prepare before transplantation^33^. Whole-mount immunostaining were done to assess in 3D image^33^. Compared to previous studies, advantages of our method are (1) simple and reproducible which no substitute are required or 2 days to prepare cell sheets before transplantation, (2) reconstruction of lymphatic vessels were observed in a short time within a week after transplantation, and (3) function of lymphatic vessels transferring hyaluronic acid are confirmed by time course not only by whole-mount immunostaining. In contrast, stability of lymphatic vessels is not evaluated in our study with longer time evaluation.

The present study evaluated peripheral lymphatics in in vivo models, and did not evaluate the potential efficacy of this method of promoting lymphangiogenesis in the field of neurology which associates with meningeal lymphatics. Therefore, additional models of disease such as stroke or dementia are required to confirm ability of transplanting these LEC-containing cell sheets onto the dura mater of a rat to evaluate potential integration with meningeal lymphatics. Additional research will also be needed to establish the optimal source of LECs for potential use in clinical practice.

## Conclusions

This is the first report to describe the formation of lymphatic vessels after the transplantation of cell sheets containing a network of LECs prepared by co-culturing LECs and ASCs. Co-culture with ASCs in conditioned medium likely exerted direct and indirect effects on LECs that promoted the formation of networks and lymphatic vessels. Although the presence of a LEC network was sufficient for construction of lymphatic vessels, cell sheets with a localized, high-density LEC network formed lymphatic vessels that were greater in number, larger in diameter and reached deeper layers. Further research is needed to explore whether the method described in this study have potential for development into new treatments for incurable neurological disorders, such as stroke, dementia and other diseases associated with lymphatic vessel impairment.

## Methods

### Measurement of growth factor levels in the culture medium bathing ASCs

ASCs (Lonza Japan, Japan) (1.0 × 10^6^ cells) were seeded on a 6-cm dish and cultured in KBM-ADSC-2 medium (16030030, Kohjin Bio, Japan) supplemented with 5% FBS. The medium was replaced with DMEM/F12 (11320033, Thermo Fisher Scientific, USA) after 3 days. After a further 3 days, the cells and medium were collected, and the cells were counted. The concentrations of VEGF-C, VEGF-A, HGF, and bFGF were measured by enzyme-linked immunosorbent assay (ELISA) using a Human VEGF-C ELISA Kit (P49767, RayBiotech, USA), LBIS Human VEGF ELISA Kit (631-40831, Fujifilm Wako, Japan), AuthentiKine Human HGF ELISA Kit (KE00168, Proteintech, USA) and Human bFGF ELISA Kit (P09038, RayBiotech, USA) in accordance with the manufacturers’ instructions.

### Evaluation of the network structure of co-cultured ECs/ASCs and co-cultured LECs/ASCs

GFP-expressing human umbilical vein ECs (Angio-Proteomie, Boston, MA, USA) and human ASCs were provided by Lonza Japan (Japan), and GFP-expressing human dermal LECs were provided by PromoCell (Germany). Co-culture of ECs and ASCs and of LECs and ASCs was performed at a ratio of 1:4 (a total of 1.0 × 10^6^ cells in each case) on a 3.5-cm dish and cultured in EBM-2 MV BulletKit (CC-3202, Lonza, Switzerland). Images were acquired using an Eclipse E800 optical microscope (Nikon, Tokyo, Japan).

### Effects of cell ratio and VEGF-C concentration on the mesh pattern of co-cultured LECs/ASCs

Human dermal LECs (PromoCell, Germany) were cultured in EBM-2 MV BulletKit (CC-3202, Lonza, Switzerland), and passages three to six were used for co-culture. Human ASCs (Lonza Japan, Japan) were cultured in KBM-ADSC-1 medium (16030020, Kohjin Bio, Japan), and passages three to six were used for co-culture. The LECs and ASCs (a total of 1.0 × 10^6^ cells) were co-cultured at a ratio of 1:4 or 3:2 in the presence of 0, 25 or 50 ng/mL VEGF-C (100-20CD-20UG, PeproTech, USA). The cells were incubated for 3 days, fixed with 4% paraformaldehyde (PFA) for 15 min, and preserved in phosphate-buffered saline (PBS) at 4 °C. Subsequently, the slides were washed with PBS followed by PBST, incubated with 0.2% Triton-X100/PBS for 5 min, and then washed three times with PBST. After blocking (Blocking One Histo, 06349-64, Nacalai Tesque, Japan) for 2 h at room temperature, the slides were incubated with anti-podoplanin/gp36 [18H5] primary antibody (ab10288, Abcam, UK; 1:50) overnight at 4 °C. On the next day, the slides were washed three times with PBST and incubated with AlexaFluor568 Goat Anti-Mouse IgG H&L secondary antibody (ab175473, Abcam, UK; 1:50) for 2 h at room temperature. Cell nuclei were stained using 4′,6-diamidino-2-phenylindole (DAPI) solution (Dojindo Laboratories, Japan; 1:200) for 15 min at room temperature. The slides were washed three times with PBST and once with PBS, and a coverslip was applied using FluorSave™ Reagent mounting medium (345789, Millipore Sigma, USA).

LEC mesh pattern was evaluated in each of the six experimental groups (n = 8 per group) by measuring the number of branches and branch lengths using a Lab-Tek II 8-Well Chamber Slide system (154534PK, Thermo Fisher Scientific, USA) and EBM-2 MV BulletKit (CC-3202, Lonza, Switzerland) as the culture medium. Nine continuous images were acquired using a confocal microscope (FV1200, Olympus, Japan), and ImageJ 2.1.0/1.53c (Bethesda, USA) was used for image analysis. The conditions for confocal microscopy were set according to the captured image. Any images that could not be evaluated (because the LEC fluorescence and background fluorescence were too bright) were disregarded; therefore, the number of images was reduced to n = 8 for the 1:4 ratio/50 ng/mL VEGF-C group, n = 8 for the 3:2 ratio/0 ng/mL VEGF-C group, n = 5 for the 3:2 ratio/25 ng/mL VEGF-C group, and n = 7 for the 1:4 ratio/0 ng/mL VEGF-C group. The images were merged by ImageJ using a stitching tool, and the number of cells was counted using the DAPI-stained images. Then, a skeletonization procedure was applied in ImageJ, and the numbers of branches and branch lengths per mm^2^ were measured.

### Preparation of cell sheets

Co-cultured LEC/ASC (1:4 ratio) sheets were prepared using a 35-mm UpCell type B dish (CellSeed, Japan), and co-cultured LEC/ASC (3:2 ratio) sheets and ASC sheets were generated using 35-mm UpCell type C dishes (CellSeed, Japan) (Fig. 4b,c). The UpCell dishes were coated with FBS for 24 h before cell seeding, and a total of 1.0 × 10^6^ cells were seeded per dish. The co-cultured LEC/ASC sheets were cultured in EBM-2 MV BulletKit medium (CC-3202, Lonza, Switzerland) containing 25 ng/mL VEGF-C (100-20CD-20UG, PeproTech, USA) for 2 days at 37 °C. The ASC sheets were cultured in KBM-ADSC-1 medium (16030020, Kohjin Bio, Japan) for 2 days at 37 °C. Subsequently, the cell sheets were harvested by incubation at 20 °C for 60 min. Three LEC/ASC (1:4 ratio) sheets were stacked together for use in the diffuse network group (Fig. 4b). Two ASC sheets were stacked sequentially on one LEC/ASC (3:2 ratio) sheet for use in the localized network group (Fig. 4c). Each three-layered cell sheet construct (Fig. 4b,c) contained a total of 0.6 × 10^6^ LECs and 2.4 × 10^6^ ASCs. From previous studies regarding cell sheet composed by temperature-responsive dish, fibronectin matrix is known to deposit on the basal surface of cell sheets. Fibronectin matrix plays a key role as an adhesive agent to promote rapid and intimate connections between grafts and hosts.

In experiments of cell sheet transplantation to a rat model of femoral lymphangiectomy (Figs. 5,6), cell sheets (localized network group) were constructed using mScarlet-I-labeled human LECs to allow the transplanted LECs to be distinguished from host LECs after transplantation in vivo. For these experiments, the LECs were labeled with mScarlet-I by lentiviral transduction. Human LECs were cultured in Endothelial Cell Growth Medium MV2 (C-22121, PromoCell, USA) for 24 h prior to lentiviral infection and were 50% confluent at the time of transduction (2.5 × 10^5^ cells/well in a 6-well plate). On the day of viral infection, mScarlet-I lentivirus (1 × 10^8^ TU/mL) was added to the cells at a concentration of 1.6 μL/well. After 24 h of incubation, the medium was replaced with Endothelial Cell Growth Medium to remove the viral particles. The lentiviral titer was confirmed using Lenti-X GoStix Plus reagent (631280, Takara Bio, Japan): 20 μL of virus diluent was applied followed by 80 μL of chase buffer, and scanning was performed after 10 min to obtain readings. Cell clones exhibiting high expression of mScarlet-I were amplified and cultured using standard methods.

### Transplantation of cell sheets or cell suspensions onto rat gluteal muscle

For transplantation of cell sheets, the skin overlying the rat gluteal region was cut and everted, and the cell sheets were transplanted onto the myofascial tissue. The cell sheets were covered with an ethylene–vinyl alcohol copolymer (EVAL) membrane (Kuraray, Japan) to prevent an inflammatory reaction or adhesion to the skin. The use of this membrane minimized inflammatory thickening of the tissues and permitted evaluation of the depth of lymphatic vessel-like structures under nearly identical conditions in every group. The EVAL membrane was stitched to the myofascial tissue at four points, and the skin was sutured.

For transplantation of cell suspensions, 0.6 × 10^6^ LECs and 2.4 × 10^6^ ASCs were collected in 100 μL of PBS (Fig. 4a). The skin of the gluteal region was incised to expose the underlying myofascial tissue. The cell suspension (Fig. 4a) was injected between the skin and myofascial tissue, and the skin was sutured at three points surrounding the region where the cell suspension had been injected (Fig. 4a).

### Evaluation of LEC-derived lymphatic vessels after the transplantation of cell sheets or cell suspensions onto rat gluteal muscle

To explore whether lymphatic vessels would develop in LEC/ASC sheets after transplantation onto rat gluteal muscle, three LEC/ASC sheets (1:4 ratio) were stacked and transplanted onto the muscle (Fig. 3). The sheets and adjacent muscle tissue were collected 2 weeks after transplantation, washed in PBS, fixed with 4% PFA and embedded in paraffin. The slides were deparaffinized, and antigen retrieval was performed using 10 mM citric acid buffer. After autoclaving for 10 min at 121 °C, endogenous peroxidase was removed by incubating the slides with 0.3% H_2_O_2_ for 15 min at room temperature. The slides were washed three times with PBST and blocked with Blocking One Histo (03953-95, Nacalai Tesque, Japan) for 10 min at room temperature. Then, the slides were incubated with anti-human podoplanin mouse monoclonal primary antibody (clone D2-40, #413451, Nichirei Biosciences, Japan; 1:10) overnight at 4 °C. The next day, the slides were washed three times with PBST and incubated with peroxidase-labeled anti-mouse IgG polyclonal secondary antibody (#424134, Nichirei Biosciences Japan; 1:200) for 30 min at room temperature. Then, the slides were washed three times with PBST and incubated with 3,3′-diaminobenzidine-4HCI (DAB) for 5 min at room temperature. The slides were washed, counterstained with Mayer’s hematoxylin (1 min at room temperature), dehydrated and mounted with a coverslip using mounting medium.

In subsequent experiments, comparisons were made between the cell suspension, diffuse network and localized network groups by evaluating the numbers, diameters and depths of lymphatic vessel-like structures constructed by the implanted LECs (n = 5; Fig. 4). One week after cell transplantation onto rat gluteal muscle, tissues were collected, washed in PBS, fixed with 4% PFA for 3 days at 4 °C, dehydrated in a graded sucrose series (10%, 20% and 30%), embedded in optimal cutting temperature compound (Sakura Finetek, Japan) and frozen on dry ice. The frozen tissues were kept at − 60 °C until sectioning was performed. Slices (8 μm) were cut using a cryostat (Leica Biosystems, Germany). For each tissue sample, twelve sections were obtained, four each of three different regions (500 μm apart) near the center of the site where cell transplantation/injection had been performed. Then, the slides were immunostained for human podoplanin as described in the preceding paragraph. Image analysis for each of the three groups was performed using three slides from different regions of each of the five samples in that group (i.e., 15 slides per group). Merged Z-stacks were acquired from three regions of each slide using confocal microscopy (FV1200, Olympus, Japan). A total of 135 images were distributed to three observers (blinded to the experimental grouping), who counted the lymphatic vessel-like structures that were tubular, positive for human podoplanin and contained nuclei. ImageJ 2.1.0/1.53c was used to measure the diameter and depth of each lymphatic vessel-like structure (depth was measured from the surface of the transplanted cell sheet to the center of the lymphatic vessel-like structure). Averaged values for each parameter were used for the analyses.

To compare relation between host lymphatic vessels and graft lymphatic vessel-like structures in three groups, immunostaining for rat LYVE-1 was also done. The slides were washed three times with PBST and blocked with Blocking One Histo (03953-95, Nacalai Tesque, Japan) for 2 h at room temperature. Then, the slides were incubated with LYVE-1 polyclonal primary antibody (#PA1-16635, Invitrogen, USA; 1:3) and anti-human podoplanin/gp36 [18H5] primary antibody (ab10288, Abcam, UK; 1:50) overnight at 4 °C. On the next day, the slides were washed three times with PBST and incubated with AlexaFluor568 Goat Anti-Mouse IgG H&L secondary antibody (ab175473, Abcam, UK; 1:50), AlexaFluor488 Goat Anti-Rabbit IgG H&L secondary antibody (ab15077, Abcam, UK; 1:50) and DAPI (Dojindo Laboratories, Japan; 1:200) for 2 h at room temperature. The slides were washed, and mounted with a coverslip using mounting medium.

### Preparation of a rat model of femoral lymphangiectomy and transplantation of cell sheets

The skin overlying the rat femur was cut and retracted, and a 1.5 × 1.5 mm piece of femoral muscle including its lymphatic vessels was resected. Then, cell sheets (prepared as for the localized network group) that had been constructed using mScarlet-I-labeled LECs were transplanted onto the defect (Fig. 5a,b). The cell sheets were covered with an EVAL membrane (Kuraray, Japan) and a silicone sheet to prevent inflammation or adhesion to the skin. The EVAL membrane was stitched to the myofascial tissue at three points, and the skin was sutured.

### Histological analysis of cell sheets transplanted in the rat lymphangiectomy model

One week after cell sheet transplantation, fluorescently-labeled (emission, 515–525 nm) hyaluronic acid (FAHA-S1; molecular weight, 40,000–80,000; PG Research, Japan) was injected into the muscle, and muscle tissue was collected after the flow of hyaluronic acid through the lymphatic vessels had been confirmed (Fig. 5i,k–m). The tissue was fixed with 4% PFA for 3 days at 4 °C and kept in PBS at 4 °C until whole-mount staining.

For tissue clearing, the sample was first incubated in 2 mL of 50% ScaleCUBIC-1 solution for 24 h at room temperature with shaking at 30 rpm. For dewaxing, the sample was incubated in 2 mL of 100% ScaleCUBIC-1 solution for 6 days at 37 °C with shaking at 30 rpm; the ScaleCUBIC-1 solution was replaced every 48 h. Then, the tissue was gently washed with PBS several times at room temperature with gentle shaking. The tissue was incubated in 0.3% Triton-X100/PBS for 30 min, washed three times with PBST, and blocked with Blocking One Histo (06349-64, Nacalai Tesque, Japan) for 2 h at room temperature. Subsequently, the tissue was incubated with anti-human podoplanin/gp36 [18H5] primary antibody (ab10288, Abcam, UK; 1:50) and anti-LYVE1 primary antibody (NB100-725F, Novus Biologicals, USA; 1:50) overnight at 4 °C. On the next day, the tissue was washed three times with PBST and incubated with AlexaFluor568 Goat Anti-Mouse IgG H&L secondary antibody (ab175473, Abcam, UK; 1:50), AlexaFluor488 Goat Anti-Rabbit IgG H&L secondary antibody (ab15077, Abcam, UK; 1:50) and DAPI (Dojindo Laboratories, Japan; 1:200) for 3 h at room temperature. After three washes with PBST and one wash with PBS, the tissue was washed with PBS at room temperature with gentle shaking and then cleared by incubation in 2 mL of 50% ScaleCUBIC-2 solution for 24 h at room temperature with shaking at 30 rpm. The sample was further incubated in 2 mL of 100% ScaleCUBIC-2 solution for 48 h at 37 °C with shaking at 30 rpm. Finally, the sample was immersed in a 1:4 mixture of Mounting Solution 1 and Mounting Solution 2.

Whole-tissue fluorescence images were acquired by two-photon microscopy. Transparent lymphatic tissue clarified with ScaleCUBIC solution was used for the 3D analysis. Microscopic images (10 × 10 × 5 mm) were acquired using a two-photon laser scanning microscopy system (FVMPE-RS Olympus, Japan) equipped with a 25 × objective lens (XLSLPLN25XGMP; numerical aperture = 1.00, working distance = 8.0 mm) and Fluoview 3.1 software. Transparent lymphatic tissue was immobilized in a container filled with SCALECUBIC solution, and the mScarlet-I signal (indicating transplanted cells), fluoro-hyaluronic acid signal (indicating perfused lymphatic vessels) and LYVE1 signal (indicating host lymphatic vessels) were detected using an excitation wavelength of 800 nm.

### Analysis of the flow of fluorescently-labeled hyaluronic acid through lymphatic vessels

Transplanted mScarlet-I-labeled LECs were observed under a stereomicroscope (MVX-10, Olympus, Japan) 1 week after cell sheet transplantation in the rat model of femoral lymphangiectomy. Fluorescently-labeled hyaluronic acid (0.2 μg in 0.02 μL PBS; FAHA-S2-1; MW, 40,000–80,000; PG Research, Japan) was injected into the femoral muscle close to the site of transplantation, and the flow of fluorescently-labeled hyaluronic acid into host lymphatic vessels was observed using a stereomicroscope (MVX10, Olympus, Japan).

### Statistical analysis

The analysis was performed using Prism 9 (GraphPad, USA). Data were compared using one-way analysis of variance (ANOVA) or two-way ANOVA with Tukey’s multiple comparison test. The level of significance was set at P < 0.05.

### Ethical approval

All animal experiments were performed in according to protocols approved by the Ethics Committee for Animal Experiment of Tokyo Women’s Medical University, and complied with the ARRIVE guidelines for the care and use of laboratory animals. The experimental protocols were approved by the Tokyo Women’s Medical University. Male F344/Jcl rnu-rnu rats (CLEA Japan, Japan) aged 8–11 weeks-old were used for the experiments. All animals were housed in individual cages with free access to food and water under a light cycle of 12 h and maintained at a constant temperature and humidity. Transplantation of cell sheets and injection of cell suspensions were performed under anesthesia (induced by 4% isoflurane and maintained by 2% isoflurane). Animals euthanasia was performed by exsanguination under 5% isoflurane according to the American Veterinary Medical Association (AVMA) euthanasia guidelines (Supplementary Video).

## Supplementary Information

Supplementary Legends.

Supplementary Video S1.

## Data Availability

The data that support the findings of this study are available from the corresponding author upon reasonable request.

## Abbreviations

ANOVA: Analysis of variance
ASC: Adipose-derived stem cell
DAPI: 4′,6-Diamidino-2-phenylindole
DMEM: Dulbecco’s modified Eagle medium
EBM-2: Endothelial basal medium-2
EC: Endothelial cell
EGM-2-MV: Microvascular endothelial cell growth medium-2
FBS: Fetal bovine serum
F12: Nutrient mixture F-12
FGF-2: Fibroblast growth factor-2
GFP: Green fluorescent protein
HB-EGF: Heparin binding-EGF-like growth factor
LEC: Lymphatic endothelial cell
LYVE-1: Lymphatic vessel hyaluronan receptor-1
NHDF: Normal human dermal fibroblasts
PBS: Phosphate-buffered saline
PBST: Phosphate-buffered saline with Tween
PDGF-BB: Platelet-derived growth factor-bb
PFA: Paraformaldehyde
Prox-1: Prospero homeobox protein-1
TGF-β1: Transforming growth factor-beta-1
VEGF-A/C/D: Vascular endothelial growth factor-A/C/D
VEGF-R1/2/3: Vascular endothelial growth factor receptor-1/2/3
