# Supplementary material for: Networked lymphatic endothelial cells in a transplanted cell sheet contribute to form functional lymphatic vessels
Source: Sci Rep. 2022 Dec 15;12:21698. doi: 10.1038/s41598-022-26041-0 (PMC9755306; doi:10.1038/s41598-022-26041-0)
Supplement: Supplementary file 1 — Supplementary Legends. [file 41598_2022_26041_MOESM1_ESM.docx]

**Supplementary material**

Video.

Fluorescently-labeled hyaluronic acid flow through the host lymphatic vessels after injection into the femoral muscle close to the site of cell sheet transplantation to the rat model of femoral lymphangiectomy. The movie was captured by a stereomicroscope (MVX-10, Olympus, Japan).
